# A new double right border binary vector for producing marker-free transgenic plants

**DOI:** 10.1186/1756-0500-6-448

**Published:** 2013-11-08

**Authors:** Jonathan M Matheka, Sylvester Anami, James Gethi, Rasha A Omer, Amos Alakonya, Jesse Machuka, Steven Runo

**Affiliations:** 1 Biochemistry and Biotechnology Department, Kenyatta University, P. O. Box 43844, 00100 Nairobi, Kenya; 2 Institute for Biotechnology Research, Jomo Kenyatta University of Agriculture and Technology, P.O. Box 62000–00100, Nairobi, Kenya; 3 Kenya Agricultural Research Institute, P.O. Box 340–90100, Machakos, Kenya; 4 Biosafety and Biotechnology Research Center, Agricultural Research Corporation, P.O. Box 126, Wad Medani, Sudan

**Keywords:** Cotransformation, Double right border, Selectable marker gene free, Removal of SMG

## Abstract

**Background:**

Once a transgenic plant is developed, the selectable marker gene (SMG) becomes unnecessary in the plant. In fact, the continued presence of the SMG in the transgenic plant may cause unexpected pleiotropic effects as well as environmental or biosafety issues. Several methods for removal of SMGs that have been reported remain inaccessible due to protection by patents, while development of new ones is expensive and cost prohibitive. Here, we describe the development of a new vector for producing marker-free plants by simply adapting an ordinary binary vector to the double right border (DRB) vector design using conventional cloning procedures.

**Findings:**

We developed the DRB vector pMarkfree5.0 by placing the *bar* gene (representing genes of interest) between two copies of T-DNA right border sequences. The β-glucuronidase (*gus*) and *nptII* genes (representing the selectable marker gene) were cloned next followed by one copy of the left border sequence. When tested in a model species (tobacco), this vector system enabled the generation of 55.6% kanamycin-resistant plants by *Agrobacterium*-mediated transformation. The frequency of cotransformation of the *nptII* and *bar* transgenes using the vector was 66.7%. Using the leaf bleach and Basta assays, we confirmed that the *nptII* and *bar* transgenes were coexpressed and segregated independently in the transgenic plants. This enable separation of the transgenes in plants cotransformed using pMarkfree5.0.

**Conclusions:**

The results suggest that the DRB system developed here is a practical and effective approach for separation of gene(s) of interest from a SMG and production of SMG-free plants. Therefore this system could be instrumental in production of “clean” plants containing genes of agronomic importance.

## Background

Removal of selectable marker genes (SMGs) from transgenic plants is increasingly becoming an important objective for the plant biotechnology research community and is viewed as a good laboratory practice. Elimination of SMGs from transgenic plants can be beneficial for the following reasons: (1) it enables the reuse of the selectable marker for identification of transformants during retransformation of a transgenic plant with a gene for the same or a different trait. (2) It allows greater probability of acceptance of transgenic plants by consumers. (3) It obviates the need to assess the SMG in the transgenic plant for environmental or toxicological safety in compliance with regulatory requirements [1].

Different techniques for elimination of selectable marker genes have been developed including some based on site-specific recombination [2] and transposition [3]. However, cotransformation stands out as a conceptually simple and cheap system to develop. Cotransformation involves introduction of a gene of interest (GOI) and SMG, harboured between separate T-DNA regions, into the plant cells. If the two transgenes integrate in unlinked genomic loci, they can be separated from each other in subsequent generation of the cotransformants through genetic segregation. Cotransformation of plants with a GOI and SMG has been achieved successfully by particle bombardment or by *Agrobacterium tumefaciens*. With particle bombardment-mediated cotransformation, the transgenes are integrated in the genome in a complex manner and rarely segregate [4]. However cotransformation mediated by *Agrobacterium* has the advantage of being capable of efficiently segregating transgenes due to the ability to integrate transgenes in a simple pattern and in few copies.

There are three main approaches used in the development of *Agrobacterium* based cotransformation systems. The most popular approach is to construct two T-DNA regions, one having the GOI and the other the SMG, on one binary vector. When introduced into *Agrobacterium*, this binary vector has exhibited high efficiency in generation of SMG-free plants very effectively [5-12]. The other common approach involves cloning the two T-DNA regions on separate binary vectors and inserting them in either one [5,13-16] or two [5,9,17-19]*A. tumefaciens* cells for use in plant transformation.

A recently developed cotransformation technique is the DRB binary vector system. This vector is a single T-DNA plasmid in which an extra copy of the RB sequence is cloned between the SMG and the GOI [20]. The general design of the DRB vector is LB-*GOI*-RB2-*SMG*-RB1. This implies that two distinct inserts may be independently transferred and integrated into the plant genome, starting either from RB1 to LB, or RB2 to LB. There exists a high possibility of a RB1 to RB2 insertion, which may significantly reduce or prevent generation of marker-free plants. Once integrated into the plant genome at unlinked locations, the second insert (RB2 to LB) which carries the GOI only can be selected for in progenies of cotransformants, while plants having the RB1 to RB2 insertion are eliminated. This DRB vector system was demonstrated to result in high cotransformation, coexpression and segregation of two transgenes in rice plants [20-22].

Over the past few years, different techniques for removal of SMGs have been developed. However, many remain inaccessible because they are protected by patents [23-26]. In addition, development of marker-removal techniques is cost prohibitive and difficult due to the large size and complexity of the vector systems. On the other hand, cotransformation vectors are, in concept, simple to develop. Most require adapting available binary vectors to the desired cotransformation vector design. Here, we report the development of a new pilot DRB vector pMarkfree5.0. It contains a selectable maker gene between RB1 and RB2. The *nptII* and *gus* gene were placed between RB2 and the LB and can be replaced with any gene(s) of interest. This binary vector was introduced into *Agrobacterium* strain LBA4404 which was used for leaf disc transformation. In addition, we report the efficient cotransfer and coexpression of transgenes contained in the different T-DNA regions in primary transformants. We also report identification of plants free of the T-DNA region containing the selectable marker gene.

## Findings

**Figure 1:**
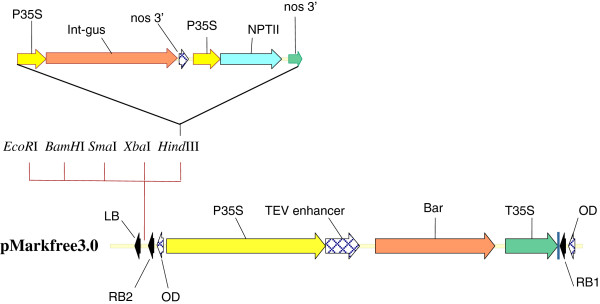
**Schematic map of the T-DNA region of the binary vector pMarkfree3.0 and insertion of the *gus *and *nptII *expression cassettes to create pMarkfree5.0.** P35S, cauliflower mosaic virus 35S promoter; T35S, cauliflower mosaic virus 35S terminator; int-gus, β-glucuronidase gene with catalase intron; nos 3’, nopaline synthase gene polyadenylation signal; LB, left T-DNA border; RB, right T-DNA border; OD, overdrive sequence.

The DRB binary vector pMarkfree3 has the structure LB-*mcs*-RB2-*bar*-RB1 (Figure 1). A multiple cloning site (mcs) comprising the *EcoR*I, *BamH*I, *Sma*I, *Xba*I and *Hind*III restriction sites is located between RB2 and LB. These restriction sites are unique to the pmarkfree3 vector and most other vectors that carry a GOI. This makes it possible to generate unique sticky ends on the DRB vector to facilitate easy cloning of any GOI. PMarkerfree3.0 vector is available on request. A DRB binary vector pMarkfree5.0 having the structure LB-*npt*II*-gus*-RB2-*bar*-RB1 (Figure 1) was developed for a quick evaluation of functionality of the DRB vector system. This could be achieved through assessment of segregation of the *npt*II*-gus* and *bar* T-DNA regions by gus staining or Basta leaf painting. The *gus* and *npt*II genes are under the control of CaMV35S promoter and the *nos* polyadenylation signal. The *bar* expression unit was composed of the CaMV35S promoter, the *bar* gene and the CaMV35S terminator.

**Figure 2:**
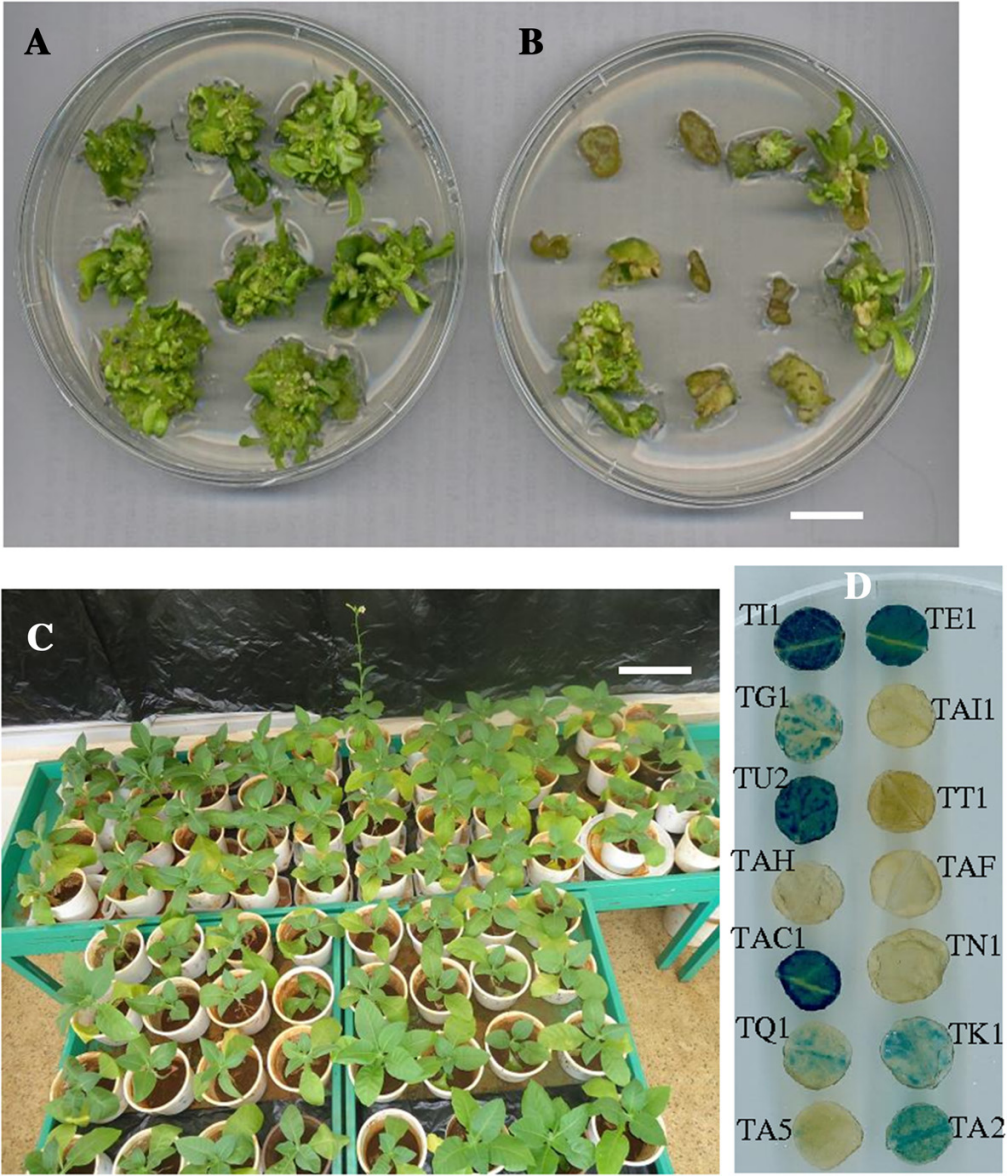
**Regeneration and establishment of putatively transformed plants.** Proliferation of shoots from putatively transformed tobacco leaf tissues after 21 days on non-selective **(A)** and selective **(B)** shoot induction medium (Bar = 1.5 cm). **(C)** Putative transgenic tobacco plants establishing in the glasshouse (Bar = 15 cm). **(D)** Histochemical staining of leaf discs to detect *gus* activity in different putatively transformed T0 tobacco plants.

Frequency of regeneration of plants was determined on explants cultured on medium with or without Kanamycin. Majority of the explants that were transformed with pMarkfree5.0 construct and placed on SM containing 100 mg/l kanamycin died (Figure 2B). On average, 56% of the explants transformed with pMarkfree5.0 survived and produced at least one shoot on kanamycin selection (Table 1). A total of 78 independent kanamycin-resistant plants were obtained from the explants surviving kanamycin selection (Table 1). These T0 plants were maintained in a greenhouse.

**Table 1:** Frequency of regeneration of plants from tobacco explants putatively transformed with pMarkfree5.0 and cultured on medium containing kanamycin (100 mg/L)

**Experiment**	**Total number of explants**	**Number of regenerating explants**
1	37	22
2	36	23
3	36	16
4	31	17
**Total**	**140**	**78**

Regeneration frequency was 55.7%, expressed as total number of regenerating explants per 100 explants infected with LBA4404 (pMarkfree5.0).

**Figure 3:**
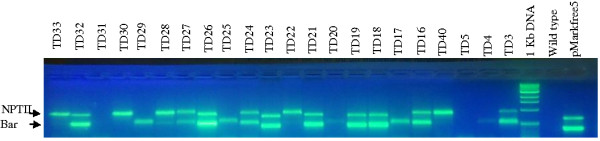
**Multiplex PCR assay for *nptII *** **and *bar *** **transgenes in transgenic T0 plants transformed using the cotransformation vector pMarkfree5.0.**

Putative transgenic tobacco plants produced using the cotransformation vector pMarkfree5.0 were analysed for the presence of the *nptII* and/or *bar* T-DNA by multiplex PCR. A representative gel image showing results of a multiplex PCR analysis for 22 putatively transformed tobacco plants is shown in Figure 3. An *nptII* PCR fragment of approximately the expected size (700 bp) was obtained from genomic DNA of 60 out of the 73 T0 plants. 66.67% of the plants that were PCR positive for *nptII* gene were found to possess a 300 bp fragment of the *bar* gene. Therefore 33.33% (20/60) of the analysed plants contained the *nptII* gene only and were consequently excluded from further analyses. Similarly plants that possessed the *bar* gene only were discarded. The first indication of a functional cotransformation system is its ability to produce the expected coinsertions. In tobacco cotransformation frequencies (CF) have varied depending on the cotransformation approach. For example CF has ranged from 59.25 [19] to 50% [18] for the two T-DNA system. With mixtures of two strains of *Agrobacterium*, CF of 20.0-47.7% [19] and 54% [17] have been reported. Daley et al. (1998) obtained a CF of 52% using two vectors in one strain of *Agrobacterium.* Evidently, about half of the primary transformants will possess multiple transgenes. Our results fall within this expectation.

Expression of *gus* gene was assessed in leaf tissues obtained from plant establishing in the glasshouse (Figure 2C) to help in rapid identification of transgenic plants. A total of 73 Kanamycin-resistant plants were screened using the histochemical assay. Figure 2D shows the gus staining results for a few of the regenerated T0 plants. Accumulation of the blue stain was observed in 47.95% (35/73) of the tested kanamycin resistant T0 plants. Although *nptII/gus* transgene was detected in 60 primary transformants by PCR, 35 of the T0 plants was the *gus* gene activity detectable. This phenomenon whereby a big proportion of transgenic plants possessing the *gus* transgene don’t show any *gus* activity by Histochemical staining has been reported elsewhere [14]. Truncation of *Agrobacterium* T-DNA region during integration in the plant genome has been used to explain this observation. We positioned the *gus* gene expression cassette closest to the T-DNA LB of pMarkfree5.0. This may have exposed it to truncation which occurs mostly at the LB [27].

**Figure 4:**
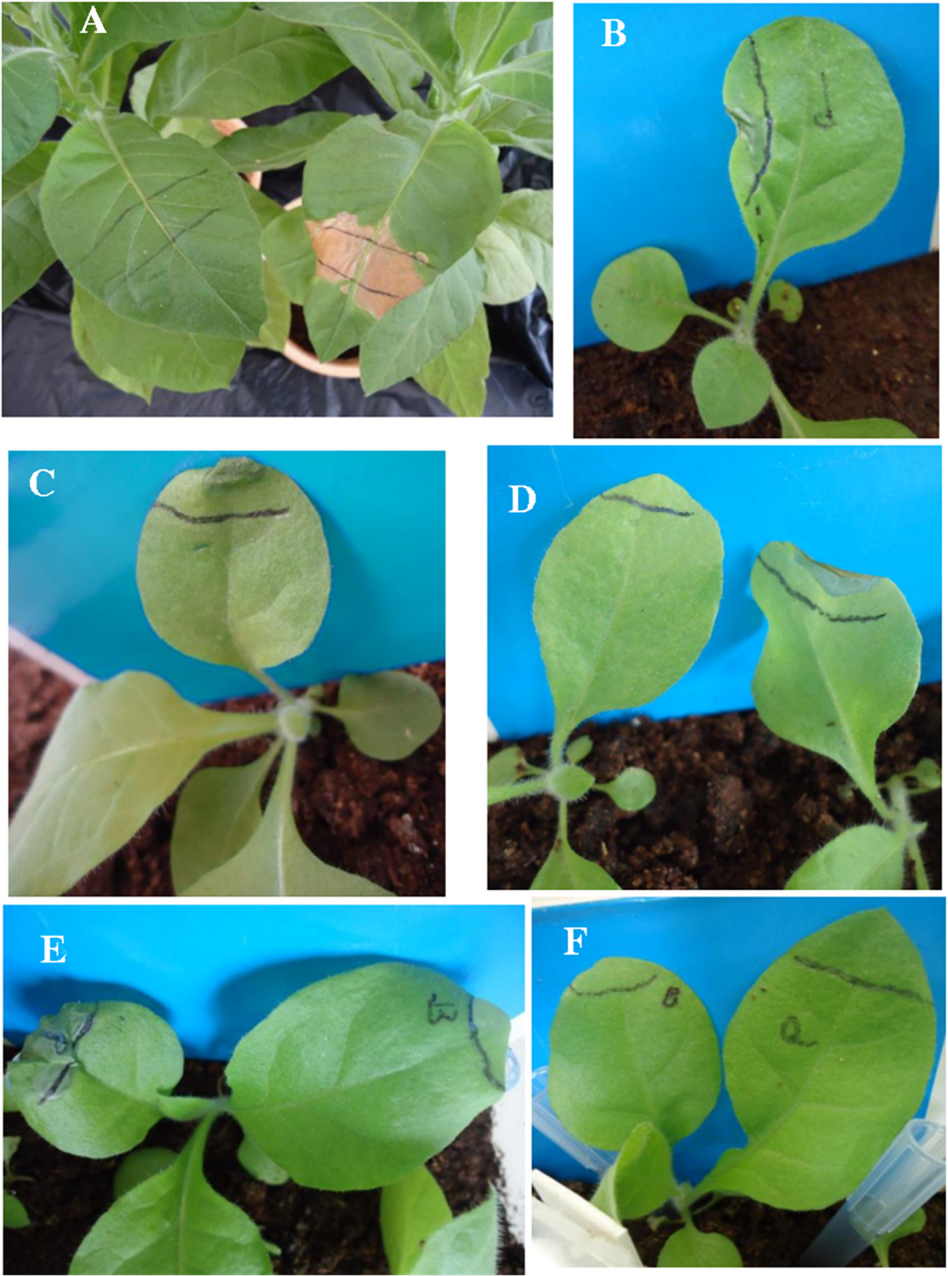
**Evaluation of expression of the *bar *and *nptII *gene in T0 and T**_**1 **_**transgenic plants using the Basta and leaf bleach assay. (A)** Effect of Basta on T0 plants. **B** and **C**: response of wildtype plants to application of leaf bleach and Basta, respectively. **D**: Effect of Basta on T_1_ progeny plants derived from the cotransformed line TD81. **E** and **F**: effect of leaf bleach on Basta resistant T1 plants derived from the cotransformed line TD81.

Stable expression of the introduced *bar* and *nptII* transgenes was confirmed by performing the Basta and leaf bleach assays on leaves of cotransformed plants. The Basta leaf paint assay on some of the cotransformed plants is shown in Figure 4A. Over 89% of the regenerated T0 plants were resistant to the application of 0.3% Basta herbicide (Table 2). Among the Basta sensitive plants were two plants confirmed to be cotransformed (event TAY3 and TAC1) by PCR analysis. These plants showed severe leaf damage even after application of relatively lower (0.02%) concentration of Basta®. The *bar* gene in the plants may have undergone silencing, truncation or rearrangement leading to its inactivity.

**Table 2:** **Functional analysis of *bar *** **and *nptII *** **genes in transformed T0 plants**

**Analysis**	**Number of plants assayed**	**Number of tolerant plants**	**Transformation frequency**
Basta leaf paint	78	70	89.73%
Kanamycin/paromomycin Leaf bleach	34	32	94.12%

Using the Kanamycin leaf bleach assay, over 94% of the primary regenerants showed no bleached spots on their leaves and were therefore kanamycin resistant (Table 2). Resistance to Kanamycin is an indication of the presence of an active *nptII* gene. Two primary transformed lines (TV2 and TAA1) had bleached spots on their leaves indicating Kanamycin sensitivity and implying absence of an active *nptII* gene. These two lines were confirmed negative for the presence of the *nptII* gene by the multiplex PCR analysis. These non-resistant plants were possibly escapes, transgenic-chimeras or could not express enough NPTII protein in their leaves to confer resistance. On the basis of the leaf bleach assay the frequency of transformation of tobacco with the *nptII* gene using the DRB pMarkfree5.0 vector was found to be 94.12%. Similar frequencies have been reported for transformation of tobacco using vectors that contain only one T-DNA region. For example a TF of 85% (Komari et al., 1996) was reported for vector pGA482. This indicates that the DRB vector is efficient in delivering transgenes into the plant genome.

Seeds from cotransformed plants were grown in soil for confirmation of out-segregation of the *bar* gene using the Basta® leaf paint assay. After screening with Basta®, all seedlings were evaluated using paromomycin/kanamycin leaf bleach assay to identify kanamycin resistant plants. Herbicide sensitive tobacco plants could be distinguished from the resistant ones three days after painting with 0.02% Basta. Seven days after application, clear effects of the herbicide were observed (Figure 4B). Application of the leaf Kanamycin/paromomycin leaf bleach solution lead to damage effects on leaves of tobacco seedlings that were similar to those of Basta (Figure 4C). Leaf damage resulting from Basta application was observed on 9 out of 19 T1 plants derived from the cotransformed line TD52. Subsequent leaf bleach test revealed leaf damage in 10 of the 19 T1 progeny plants assayed. These phenotypic assay results confirmed that the *bar* and *nptII* transgenes were segregating in the T1 plants. Leaf damage developed on 4 out of 19 T1 seedlings derived from the cotransformed line TD81 while the rest were completely resistant to the damaging effect of Basta (Table 3). The same cotransformed line TD81 failed to segregate kanamycin resistance to its progenies. This is because all of the T1 progeny plants derived from cotransformed line TD81 contained a functional *nptII* gene (Table 3).

**Table 3:** Segregation of Basta and kanamycin resistance in T1 plants derived from cotransformed plants

**T0 event**	**Number of T1 plants assessed by Basta leaf painting**		**Number of T1 plants analysed by paromomycin Leaf bleach assay**		**Number of T1 plants**				**Frequency of generation of marker-free plants**
	**B**^**R**^	**B**^**S**^	**Km**^**R**^	**K**^**S**^	**B**^**R**^**Km**^**R**^	**B**^**R**^**Km**^**S**^	**B**^**S**^**Km**^**R**^	**B**^**S**^**Km**^**S**^	
TD56	15	8	15	8	15	0	0	8	0.00%
TD81	15	4	19	0	15	0	4	0	21.05%
TD52	8	11	9	10	6	4	3	6	15.79%

B^R^, Basta resistant; B^S^, Basta sensitive; Km^R^, kanamycin resistant; Km^S^, kanamycin sensitive.

Our results indicate that the frequency of removal of marker gene from transgenic T1 plants was between 0 and 40%. Similarly, Hong-Yan et al. (2003), using a DRB system, observed that 19.5% of T1 plants derived from tobacco plants cotransformed with *nptII* and *bar* transgenes were free of the *nptII* gene. Therefore recovery of SMF T1 plants from T0 plants cotransformed using pmarkfree5.0 is highly efficient.

**Figure 5:**
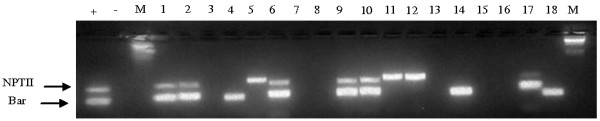
**Multiplex PCR analyses for the presence of *bar *and *nptII *in 19 T1 progeny plants derived from cotransformed line TD52. Lane +: genomic DNA from TD52 line used as positive control.** Lane -: non-template control. Lane M: HindIII/lambda DNA. Lane 1–18: T1 plants derived from the cotransformed line TD52.

Absence of the *bar* gene from progeny plants derived from cotransformants was confirmed by molecular analyses. T1 Progeny plants derived from the cotransformed line TD52 exhibited the 700 bp *nptII* gene in 9 plants. Of these, 6 revealed the presence of a 300 bp bar gene fragment. Therefore in 3 of the progeny plants, the *bar* gene was absent (Figure 5). This indicates that the *bar* gene was not inherited in these three progenies and were therefore marker-free. PCR conducted on 19 progeny plants of TD81 revealed the presence of a 300 bp fragment of the *bar* gene in 12 of the plants. However, the 700 bp band diagnostic of the *nptII* gene was present in all the 19 progenies assayed. The presence of the *nptII* gene in all the T1 plants suggests that it was not segregating. This is because of existence of more than once copy of the nptII gene in the T0 event TD81. However, the bar gene segrated in the T1 plants, indicating its existence as a sigle copy in the parental plant TD81.

PCR analyses also revealed that all T1 progeny plants derived from line TD56 were either negative or positive for both the *bar* and *nptII* T-DNA insertions. This indicates absence of genetic separation between the *bar* and *nptII* T-DNAs regions. This means that the integration of the two T-DNA regions was in the same genomic location in the cotransformants making the two inserts segregate together. Therefore no marker-free progeny plants were produced by the parental line TD56.

Successful genetic separation of two T-DNA is dependent on various factors. One of the most important factor seems to be strain of *A. tumefaciens* used to deliver the multiple T-DNAs. Nopaline-derived *A. tumefaciens* strains may favour insertion of multiple T-DNA in linked genomic loci. Initially, when two distinct T-DNAs were separately inserted in two nopaline-derived *Agrobacterium*, marker-free *Arabidopsis thaliana* or *Brassica napus* were produced at a very low frequency [28-30]. Recently, the frequency of generation of maker-free plants increased when the same strain (EHA101) or its derivative (EHA105) was used in combination with a two T-DNA vector [8,12]. This implies that differences in the plant species and strain/vector used could alter genetic linkage relationship.

Currently cotransformation systems are mainly based on octopine-derived *Agrobacterium* strains possibly because they may favour unlinked transfer of independent T-DNAs [11]. Among the octopine-derived *Agrobacterium* strains, LBA4404 is the most popular. Using this strain high efficiencies of marker-free plants have been obtained from different species including barley [5,10], maize [9,31], rice [18] and tobacco [11,15,17-19]. Apart from the *Agrobacterium* strain used, the type of the T-DNA borders regions on the cotransformation vector may affect unlinked transfer of multiple T-DNAs.

## Conclusion

We have developed a new DRB binary vector, pMarkfree3.0 and inserted the *gus* and *nptII* genes to create the model vector pMarkfree5.0. Using the model vector we have demonstrated the functionality of the new system in that: 1) we have generated over 70 independent transgenic tobacco plants. 2) Over 66% of our transgenic plants contained two transgenes (*nptII* and *bar*) originating from different T-DNA regions. Over 89% of these plants co-expressed the two genes. 3) The transgenes in 50% of the plants were inherited as Mendelian traits, making it possible to recover marker-free plants through segregation. This demonstrates that the newly developed DRB system is an effective way to remove undesirable genes from the plant genome. In ongoing research projects at the Plant Transformation Laboratory, Kenyatta University, genes of agronomic interest including those conferring tolerance to drought are being inserted into pMarkfree3.0 for transformation of crops such as maize and sweetpotato. We are also developing and using two T-DNA binary vectors that, in principle and design, are similar to the pMarkfree3.0 to engineer maize for enhanced tolerance to drought.

## Methods

### Construction of DRB binary vectors

The DRB vectors were constructed in the backbone of the binary vector pSCV1.6 [32]. The pSCV1.6 plasmid was digested with *Hind*III to delete the *gus* and *npt*II gene cassettes from the T-DNA region. The resultant vector (pSCV∆*NPTIIGUS*) was then self-ligated using T4 DNA ligase. The T-DNA region of the plasmid pSCV∆*NPTIIGUS* was amplified and subcloned into the *Not*I/*Cla*I sites of pBluescript(SK-) (Stratagene, Cambridge, UK) to produce the new vector pBlu2SK::EmptyTDNA. PTF101.1 [33] was digested by *Bgl*II and *Hind*III to excise the P35SBar fragment. The fragment was then ligated with pBlu2SK::EmptyTDNA vector pre-digested with *Bgl*II and *Hind*III to produce the new vector pBluTDNA::P35SBar. PBluTDNA::P35SBar was digested by *Bgl*II and purified. Purified DNA was end-filled using DNA polymerase I, large (klenow) fragment. The blunted DNA was dephosphorylated with Alkaline phosphatase, calf intestinal (CIP). CIP’d DNA was ligated with a T35S termination sequence amplified by PCR on pXBb7-SI-UBIL plasmid [34]. Ligation mixture was then used to transform competent *E. coli* cells. DNA extracted from selected colonies was screened using a vector-specific and an insert-specific primer to ascertain the orientation of the T35S insert. The vector with the correct T35S insertion was named pBluBarTDNA. PSCV∆*NPTIIGUS* vector, digested with *Xho*I, was end-filled and CIP’d. The *bar* expression cassette flanked by a copy of the right border sequence was removed from pBlubarTDNA as an *Asc*I*/Hind*III fragment. The fragment was end-filled and ligated onto the CIP’d and blunted pSCV∆*NPTIIGUS* vector. Ligation products were transformed into competent *E. coli* cells. DNA from emerging colonies were screened by digesting with *Kpn*I to determine the orientation of the fragment inserted in the pSCV∆*NPTIIGUS* vector. The correct vector was named pMarkerfree3 and underwent sequencing to confirm the directionality of the T-DNA border sequences. The *Sal*I*/Bgl*I fragment haboring the *gus* and *npt*II gene cassettes was removed from pSCV1.6 and end-filled. The fragment was then subcloned into pMarkfree3 predigested with *Hind*III to produce pMarkfree5.0. PMarkfree5.0 was mobilized into the *A. tumefaciens* strain LBA4404 [35] using the freeze thaw technique [36]. The new *Agrobacterium* strain was cultured on yeast extract-mannitol (YM) medium [37] containing rifampicin (1 mg L^-1^) and kanamycin (50 mg L^-1^).

### Transformation of tobacco with pMarkfree5

Leaf discs of *Nicotiana tabacum* were transformed using the cocultivation method [38]. Transformed shoots were regenerated on MS medium [39] containing B5 vitamins [40]. The medium also contained 100 mg/l kanamycin for transgenic plant selection and 200 mg/l timentin for suppression of *A. tumefaciens* growth. Regenerated shoots were separated from leaf tissues and were rooted on hormone-free MS basal medium containing 3% sucrose and 200 mg/l timentin. Plants were maintained in culture at a 16 h/8 h light/dark photoperiod at 27°C. Rooted shoots were transfered to soil for seed set.

### Screening of tobacco cotransformants by multiplex PCR

Genomic DNA was extracted from tobacco leaf tissues using the CTAB method [41]. 250–20 ng of the DNA was used in PCR to detect the presence of *bar* and *nptII* genes. Each 50 ul reaction contained 1X PCR reaction buffer containing 3 mM MgSO_4_, 0.5 μM each primer, 1 mM of dNTP mixture and 2U Taq DNA polymerase. The primers used for amplification of the *bar* gene were Bar-fwd: 5’ gatctcggtgacgggcagga 3’ and Bar-rev: 5’ ggtcaacttccgtaccgagc 3’. Primers for amplification of *npII* gene were NPTII-forward: 5’ ggattgcacgcaggttctc 3’, NPTII-reverse: 5’ ctcttcagcaatatcacgggt 3’. The PCR profile involved initial denaturation at 94°C for 10 minutes, followed by 35 cycles of denaturation at 94°C for 3 minute, annealing at 63°C for 1 minute and extension at 72°C for 1 minute with a final extention at 72°C for 8 min. Amplicons were visualised in a 1.5% agarose gel and results documented using a digital camera.

### Basta leaf paint assay

To assess functionality of the *bar* gene, Basta® leaf paint assay [42] was applied on cotransformed plants. The plants were swabbed with Basta (0.02%) applied using a piece of cotton wool to a small section of a tobacco leaf. The section of leaf to be painted with Basta was first marked using a permanent marker pen. After seven days, putative transgenic tobacco plants were scored for response to the applied herbicide. Plants that showed no leaf damage were classified as Basta resistant (B^R^) while those that were damaged were classified as Basta sensitive (B^S^).

### Leaf bleach and histochemical assays

The leaf bleach assay was performed on plants growing in soil to identify those containing a functional *nptII* gene. The assay was performed by applying assay solution containing paromomycin (Duchefa Biochemie B.V., Haarlem, The Netherlands) and kanamycin (Phytotechnology) each at 1,000 mg/l and 0.06% of Silwet L-77 (Lehle Seeds, Texas, USA) on a small section of a leaf using a piece of cotton wool. Results were recorded on the 7th day post application. Plants that showed no bleaching were categorized as kanamycin resistant (Km^R^) while those that bleached were categorized as kanamycin sensitive (Km^S^). Histochemical assay for β-glucuronidase (GUS) activity were performed on leaf tissues as described previously [43].

### Phenotypic and molecular assays of T1 seedlings

To identify cotransformed lines that were segregating the *bar* gene, T1 seedlings were screened for resistance to PPT. About 100 T1 seeds were sown on MS medium containing 10 mg/l PPT. 14 days later, survival of tobacco seedlings to PPT was examined. Plants that were green and growing vigorously were categorized as PPT-resistant (PPT^R^) while those that were small and bleached were classified as PPT-sensitive (PPT^S^).

To identify plant free of the *bar* gene, T0 events with single-copy of *bar* gene were selected for T1 segregation analysis. T1 seedlings were germinated on MS medium and transplanted to soil. Once established, the plants were assayed for resistance to Basta (0.02%) as described previously. The leaf bleach assay was performed on T1 seedlings previously assayed for Basta resistance to identify plants expressing the *nptII* gene. The marker-free plants identified based on phenotypic assays (Basta and kanamycin resistance) were advanced for confirmation through PCR as described previously.

### Data analysis

Chi-square goodness-of-fit tests were performed on data from the T1 populations derived from self pollinated cotransformed tobacco plants to determine if the observed segregation ratios of PPT, Basta or NPTII resistant plants to PPT, Basta or NPTII sensitive plants fit the expected mendelian 3:1 or 1:1 phenotypic ratios, respectively.

## Availability of supporting data

All the supporting data are included as additional files.

## Abbreviations

DRB: Double right border; SMG: Selectable marker gene; GOI: Gene of interest; PPT: Phosphinothricin; PCR: Polymerase chain reaction.

## Competing interests

The authors declare that they have no competing interests.

## Authors’ contributions

JMM designed and carried out all experiments, participated in the interpretation of the results and wrote the first draft of the manuscript. S.A participated in the design of the study, interpretation of the results and drafting of the manuscript. J.G participated in the design of the study, interpretation of the results and drafting of the manuscript. S.R participated in vector development, PCR, interpretation of the results and drafting of the manuscript. R.A.O participated in vector development, DNA extraction and drafting of the manuscript. A.A participated in the design of the study, vector development, interpretation of the results and drafting of the manuscript. J.M conceived the study, participated in the experimental design and coordination, interpretation of the results and drafting of the manuscript. All authors read and approved the final manuscript.
